# First steps of laparoscopic surgery in a sub-Saharan African setting: a nine-month review at the Douala Gynaeco-Obstetric and Pediatric Hospital (Cameroon).

**Published:** 2017-06

**Authors:** JT Fouogue, FY Fouelifack, JH Fouedjio, R Tchounzou, Z Sando, ET Mboudou

**Affiliations:** Gynaeco-Obstetric unit of the Douala Gynaeco-Obstetric and Pediatric Hospital; Douala – Cameroon. PO Box 812.; HigherInstitute of Medical Technologies; Yaoundé – Cameroon. PO Box: 31186.; Faculty of Medicine and Biomedical Sciences of the University of Yaoundé 1; Yaoundé – Cameroon. PO Box: 1364; Faculty of Health Sciences of the University of Buea; Buea – Cameroon. PO Box 63.

**Keywords:** Laparoscopy, Cameroon, endoscopy, surgery, gynaecology, minimal invasive, resource-poor countries

## Abstract

**Objectives:**

To describe the first laparoscopic surgeries in a tertiary hospital in Cameroon.

**Methods:**

We carried out a descriptive study at the Douala Gynaeco-Obstetric and Pediatric Hospital (DGOPH). We examined the files of the 45 patients who underwent laparoscopic surgery from November 1, 2015 to July 31, 2016. Descriptive statistics were computed for patients’ characteristics and surgical parameters.

**Results:**

Mean (SD) age was 36.8(11.9) years. Women made up 86.7% of the sample. Twelve patients (26.7%) had a previous laparotomy. Cash deposit (86.7%) was the main mode of payment. Thirty-two (71.1%) laparoscopies were gynaeco-obstetrical (GO) and 13 (28.9%) were digestive. Main indications were infertility (59.4%) and chronic cholecystitis (30.8%) for GO and digestive laparoscopies respectively. Mean (SD) durations were 89.1(57.5) and 55.5(41.0) minutes for digestive and GO laparoscopies respectively. Mean (SD) costs were 1065.4 (406.1) and 934.2 (657.0) USD for digestive and GO laparoscopies respectively. Mean (SD) lengths of hospital stays were 5.5 (2.5) and 5.5 (2.5) days for digestive and GO laparoscopies respectively. Local staff carried out all GO laparoscopies while foreign (Belgian) experts did digestive cases. Only one (2.2%) complication (colic perforation) was registered.

**Conclusion:**

The beginnings of laparoscopy at the DGOPH were successful thanks to strong local leadership and Belgian technical assistance.

## Introduction

Since its popularization about three decades ago, laparoscopy has revolutionized the practice of surgery in developed countries (Gyedu et al., 2015; Alan, 1997; Ekwunife and Nwobe, 2014). Classically referred to as “minimal invasive” surgery it has evolved to “minimal-access” surgery because several advanced and invasive procedures are nowadays carried out by laparoscopy (Gyedu et al., 2015; Alan, 1997). This is true for both digestive and gynaecologic surgery. For instance, laparoscopy has become the gold standard for cholecystectomy, cephalic duodenopancreatectomy, appendectomy, ectopic pregnancy and benign adnexal masses (Gyedu et al., 2015; Owono, 2008).

Despite its numerous advantages over laparotomy (less trauma to tissues, less postoperative pain, shorter hospital stay, earlier duty resumption, less postoperative adhesions, better cosmesis, less blood loss, lower overall costs), laparoscopic surgery is not wide-spread in low- and middle-income countries (LMICs) (Gyedu et al., 2015; Ekwunife and Nwobe, 2014; Owono, 2008; Chao et al., 2016; Amin et al., 2015; Akintobi et al., 2015). Barriers to the spread of laparoscopic surgery in LMICs have been grouped into three categories beyond the lack of funding for costly equipment and expertise usually evoked (Choy et al., 2013). The first category is about inadequate strategy of funding that limits the number of laparoscopic cases; the second category is about the hierarchical nature of surgical practices that doesn’t promote adoption of new technology; the third category is about attitudes of surgeons who are less willing to carry out more technically complicated and time-consuming procedures (Choy et al., 2013).

**Figure 1:**
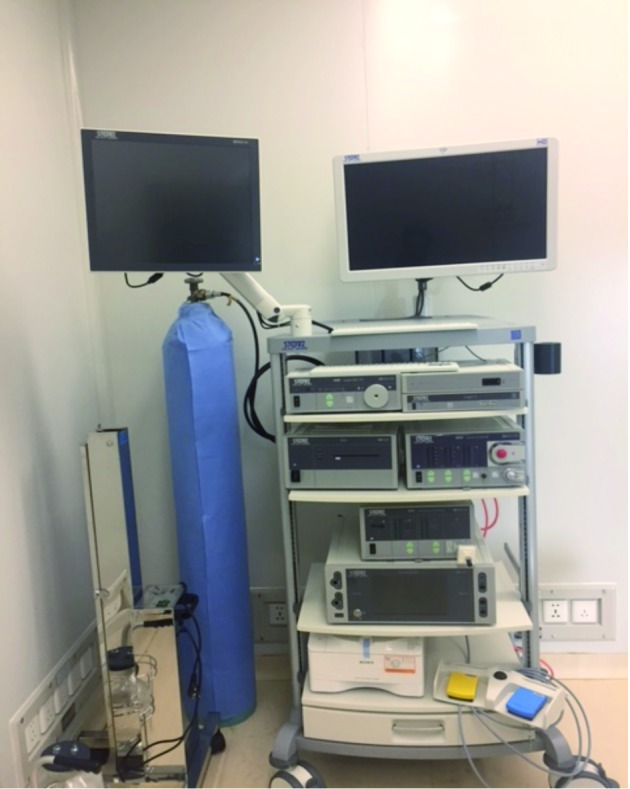
— A view of the laparoscopy unit used during the study period

In spite of the above-mentioned barriers, laparoscopic surgery has slowly but surely been established in several resource-poor African settings (Gyedu et al., 2015; Gyedu et al., 2015; Owono, 2008; Chao et al., 2016; Amin et al., 2015; Akintobi et al., 2015; Arung et al., 2015; Camara et al., 2015; Bedada et al., 2015; Diop et al., 2008; Casanelli et al., 2007). In Cameroon, since its initiation by the pioneer team of the Yaoundé General Hospital in 1992, laparoscopic surgery has wide-spread and is nowadays practiced in at least six public and eight private settings located in Douala and Yaoundé (economic and political capitals respectively) (Kasia et al., 2016; Raiga et al., 1994; Mboudou et al., 2013; Tchente et al., 2009; Nzintcheu et al., 2012; Kemfang et al., 2015). The Douala Gynaeco-Obstetric and Pediatric Hospital (DGOPH) is the largest and the most recent centre (started in September 2015) in Cameroon dedicated to mother and child care. A laparoscopic surgery programme was started at the DGOPH in November 2015. This was a major item of the road map of the top management towards efficient and accessible laparoscopic surgery. A basic unit of laparoscopy (Figure1) was acquired by the hospital on capital funds (Karl Storz GmbH & Co. KG - Tuttlingen, Germany). A training programme was started with the support of Belgium (institutional back up, expertise and funding for training Cameroonians surgeons abroad and locally, supply of additional devices for complex surgeries). At the beginning, three physicians (two gynaecologists and one urologist) were admitted for a one year internship in Belgium while two proficient gynaecologists aided by two Belgian surgeons started laparoscopies at the DGOPH. Maintenance of the laparoscopy unit was ensured by the supplier (after-sales service). After 9 months of practice, an evaluation was necessary in view of ensuring quality control as per recommendations by international organisations (Mendes Da Costa, 2016). We therefore undertook to review the first cases.

## Methods

We carried out a cross-sectional and descriptive study at the Douala Gynaeco-Obstetric and Pediatric Hospital (DGOPH). Laparoscopic surgery was started in November 2015 in that tertiary and teaching hospital located in the economic capital of Cameroon (Central Africa). We included medical records of all patients who underwent laparoscopic surgery from November 1, 2015 to July 31, 2016. Incomplete records were excluded. Prior to data collection a clearance was obtained from the institutional ethics committee of DGOPH (N °001/AR/HGOPED/DG/ DM/SRFESS/pt). Confidentiality was observed. Informed consent was not necessary because only hospital records were exploited. The following parameters were collected: age, sex, gestity and parity (for women), marital status, referral status, mode of payment, previous abdomino-pelvic surgery (laparoscopy or laparotomy), pregnancy status during surgery, indication of laparoscopy, duration of the surgery, length of service of the surgical team, surgical findings, date of surgery, cost of the procedure, length of hospital stay, conversion to laparotomy and complications. Data management was done with Microsoft Office Excel® (version 2010) software.

## Results

During the study period, 45 laparoscopic surgeries were done.

### Characteristics of patients

Table I describes the characteristics of patients. Mean age was 36.8 +/- 11.9 years. One of the 39 (2.6 %) women had intra-uterine pregnancy (19 weeks and 3 days) during laparoscopy (indicated for ruptured haemorrhagic ovarian cyst). Twelve of our patients (26.7%) have had previous laparotomy and 5 (11.1%) have had previous laparoscopy. Referred patients made up 31.1 % (14 out of 45) of our sample. The financing was done by cash payment for 39 (86.7%) patients and by health insurance companies for 6 (13.3%) of patients.

**Table I:** — Characteristics of patients (n = 45)

**Characteristics**	**Frequencies**	**Proportions (%)**
**Age (years)**		
≤ 15	1	2.2
16 – 30	11	24.4
31 – 45	25	55.6
≥ 46	8	17.8
**Sex**		
Male	6	13.3
Female	39	86.7
**Gravidity**^f^ 0 – 1	17	43.6
2 – 4	19	48.7
≥ 5	3	7.7
**Parity**^f^ 0	21	53.8
1 – 5	18	46.2
**Marital status**		
Married	31	68.9
Single	13	28.9
Widow (er)	1	2.2
**Occupational status**		
Student	3	6.7
Unemployed	9	20.0
Employed	32	71.1
Retired	1	2.2

f : female patients only

### Characteristics of laparoscopic surgeries

Table II shows the characteristics of laparoscopic surgeries. Local staff carried out all the gynaeco-obstetrical laparoscopies (32/45; 71.1%). They were all carried out by the same obstetrician-gynaecologist (having 25 years of experience in laparoscopy) assisted in 62.5 % (20/32) of them by the same doctor (obstetrician-gynaecologist having 2 years of experience in laparoscopy). Foreigners did all digestive (13/45; 28.9%) in the frame of a training and exchange programme between the DGOPH and several institutions in Belgium (During two editions of 6-day training workshops in November 2015 and June 2016). Of the 32 laparoscopies carried out by local staff, anaesthesia was done by the same doctor (with 3 years of experience in laparoscopy) in 75 % (24/32) of cases. Only 3 (6.67%) laparoscopies were emergency procedures. No conversion to laparotomy was done. One (2.2%) complication was registered: a perforation of the sigmoid colon during laparoscopic hysterectomy (with subsequent peritonitis).

**Table II:** — Characteristics of laparoscopic surgeries (n = 45)

**Indications**		**Frequencies (%)**	**Mean (SD) duration (minutes)**	**Mean (SD) cost (USD)**	**Mean Length (SD) of hospital Stay (Days)**
**Digestive**		**13 (28.9)**	**89.1(57.5)**	**1065.4 (406.1)**	5.5 (2.5)
	Chronic cholecystitis	4 (8.9)	77.3 (21.1)	1184.9 (608.7)	4.6 (0.5)
	GIT tumour	3 (6.7)	118.7 (121.7)	1101.6 (550.9)	8.3 (4.2)
	Ascites of unknown origin	1 (2.2)	55 (NA)	649.1 (NA)	3 (NA)
	Hiatal hernia	1 (2.2)	120 (NA)	765.6 (NA)	4 (NA)
	Pancreatic tumour	1 (2.2)	70 (NA)	1146.1 (NA)	4 (NA)
	Pathologic GER	1 (2.2)	109 (NA)	1070.0 (NA)	6 (NA)
	Peptic pyloric stenosis	1 (2.2)	100 (NA)	910.9 (NA)	6 (NA)
	Symptomatic biliary sludge	1(2.2)	39 (NA)	1094.4 (NA)	5 (NA)
**Gyneco-Obstetrical**		**32 (71.1)**	**55.5 (41.0)**	**934.2 (657.0)**	5.2 (5.9)
	Infertility	19 (42.2)	47.6 (19.9)	781.1 (291.3)	3.8 (0.5)
	Secondary infertility	14 (31.1)	50.4 (20.7)	829.6 (303.9)	
	Primary infertility	5 (11.1)	39.8 (17.1)	645.3 (224.8)	3.6 (0.5)
	Post-myomectomy	5 (11.1)	65.3 (42.8)	787.1 (122.8)	3.9 (0.5)
	Non ruptured ectopic pregnancy	2 (4.4)	42.5 (3.5)	985.3 (96.0)	3 (1.4)
	Organic ovarian cyst	2 (4.4)	40.0 (7.1)	878.4 (13.6)	3.5 (0.7)
	Chronic pelvic pain	2 (4.4)	40.0 (21.2)	779.7 (26.9)	4.5 (0.7)
	CIN 3	1 (2.2)	235 (NA)	4203.6 (NA)	36 (NA)
	Ruptured hemorrhagic ovarian cyst	1 (2.2)	150 (NA)	1627.8 (NA)	15 (NA)

USD (symbol): United States Dollar; SD: Standard Deviation; NA: Not Applicable; CIN: Cervical Intra-epithelial Neoplasia; GIT: Gastro-Intestinal Tract. GER: GastroEsophageal Reflux

### Laparoscopic findings in infertility

Findings during laparoscopies indicated for infertility are detailed in table III. Prevalence of peri-hepatic adhesions was 26.3% while pelvic adhesions and tubal obstruction were found in 57.9% of cases.

**Table III:** — Laparoscopic findings in infertility (n=19)

**Laparoscopic findings**		**Frequencies**	**Proportions (%)**
Pelvic adhesions		11	57.9
Tubal obstruction		11	57.9
	- Unilateral tubal obstruction	8	
	- Bilateral tubal obstruction	3	
Uterine abnormalities		9	47.4
	- Adenomyosis	2	
	- Myomes	6	
Peri-hepatic adhesions (Fitz-Hugh-Curtis syndrome)		5	26.3
Salpingitis isthmica nodosa		4	21.1
Ovarian abnormalities		4	21.1
	- Dystrophy	1	
	- Functional cyst	3	
Endometriosis		4	21.1
Bilateral Tubo-ovarian abscess		1	5.3

## Discussion

Carrying out forty-five laparoscopies over nine months in a tertiary centre is few in the strict sense. Nevertheless, the DGOPH was at its debut and two other factors could explain the low frequency of laparoscopic surgeries: the shortage of staff proficient in laparoscopic surgery and the relatively high costs. Indeed, all the gynaeco- obstetric laparoscopies were carried out by the same local obstetrician/gynaecologist while all digestive surgeries were done by foreigner because none of the local visceral surgeons was qualified in laparoscopy. To date laparoscopy doesn’t have enough space in the training curricula of residents in Cameroon. Therefore regular training workshops by foreign experts (Belgians in the case of the DGOPH) targeting certified gynaecologists and surgeons is a short-term solution that has been successful in several low-and middle-income countries (Gyedu et al., 2015; Asbun et al., 1996). The sustainable solution is to implement of a low- cost laparoscopic skills curriculum in post-grade medical and nursing schools in the country (Long et al., 2014).

Average costs (934.2 (+/- 657.0) and 1065.4 (+/- 406.1)) USD were very high for patients in a lower to middle-income country like Cameroon (The World Bank Group, 2016). Though direct cash payment is the most inefficient mode of purchasing health services, 86.7% of our patients paid by cash deposit because of the poor health insurance coverage in Cameroon (1%) (Nkoa and Ongolo-Zogo, 2012). Despite the fact that overall costs of laparoscopic surgery are lower than those of classic surgery, poor financial access and inadequate funding structure are commonly reported in studies from low- and middle-income countries (Chao et al., 2016, Choy et al., 2013). Popularization of the supply of laparoscopic surgery in Cameroon may reduce its costs and foster access by the population.

There was only one paediatric patient in our sample and female patients predominated (86.7%).

This is because the study took place in a setting (DGOPH) dedicated to women and child health without a paediatric surgeon. Seventy-one per cent of our patients were employed which is consistent with the economic capital in which the study took place. The mean age of patients in our series was similar to those reported in other African countries but mean lengths of hospital stay were longer (Ekwunife and Nwobe, 2014, Camara et al., 2015, Casanelli et al., 2007). The novelty of the practice in laparoscopy in our setting is certainly the reason.

Digestive laparoscopies in our series were complex procedures (carried out by foreign partners) while gynaeco-obstetric (carried out by local doctors) were simple, belonging the second level of the European Society for Gynaecological Endoscopy classification (Campo et al., 2012). This explains the big difference in mean durations of procedures (89.1(+/- 57.5) versus 55.5 (+/- 41.0) minutes). This denotes a need for capacity building via further training. Nevertheless, no conversion to laparotomy was done and we had fewer complications (2.2%) than reported in neighbouring countries (Ekwunife and Nwobe, 2014, Casanelli et al., 2007). An explanation is that laparoscopies in this series were carried out by foreign internationally reputed surgeons and by local experienced gynaecologists-obstetricians.

In our series, infertility was mainly secondary (73.7%). Similar findings were reported elsewhere in Cameroon by Kemfang et al. (71.6%), Kasia et al. (70.1%) and Mboudou et al. (72.7%) (Kasia et al., 1994; Mboudou et al., 2013; Kemfang et al., 2015). Findings during laparoscopies for infertility in our series were dominated by pelvic adhesions (57.6%) and tubal obstruction (57.6%), which are common in infertile black African women. Kasia et al. found 83.3% of pelvic adhesions while Mboudou et al. reported 71.6% of pelvic adhesions in infertility (Kasia et al., 1994; Mboudou et al., 2013). Globally, tubal abnormalities accounts for 40% of female subfertility (Steinkeler cited by Kasia) (Kasia et al., 1994). The prevalence of peri-hepatic adhesions in our study (26.3%) was similar to those previously reported in the country (Mboudou et al., 2013; Nzintcheu et al., 2012; Kemfang et al., 2015).

Studies reporting the beginnings of laparoscopy in Cameroonian settings are rare and limited to the political capital (Yaoundé) (Kasia et al., 2016; Raiga et al., 1994; Mboudou et al., 2013; Kemfang et al., 2015). Our study was carried out in Douala (economic capital) and illustrates how strong local commitment together with foreign technical assistance can establish laparoscopic surgery in low- and middle-income countries. Contrary to prejudices, maintenance of laparoscopic material was successfully realised in our resource-poor setting. However the smallness of our sample size is a weakness. It will be worthwhile to conduct long-term follow-up studies at the DGOPH.
